# A Survey of Anxiety and Depressive Symptoms in Pulmonary Tuberculosis Patients With and Without Tracheobronchial Tuberculosis

**DOI:** 10.3389/fpsyt.2018.00308

**Published:** 2018-07-19

**Authors:** Xiao-bo Wang, Xue-lian Li, Qing Zhang, Juan Zhang, Hong-yan Chen, Wei-yuan Xu, Ying-hui Fu, Qiu-yue Wang, Jian Kang, Gang Hou

**Affiliations:** ^1^Department of Respiratory Medicine, The First Hospital of China Medical University, Shenyang, China; ^2^Department of Epidemiology, School of Public Health, China Medical University, Shenyang, China; ^3^Department of Endoscopy, Shenyang Thoracic Hospital, Shenyang, China; ^4^Department of Laboratory, Shenyang Thoracic Hospital, Shenyang, China; ^5^Department of Respiratory Medicine, Anshan Central Hospital, Anshan, China; ^6^The Fourth Department of Tuberculosis, Shenyang Thoracic Hospital, Shenyang, China

**Keywords:** tuberculosis, social support, mental health, depression, anxiety, tracheobronchial tuberculosis

## Abstract

**Background and Objective:** Anxiety/depression and tuberculosis are global public health problems. The incidence of psychiatric morbidities is high among tuberculosis patients. However, little is known about the prevalence of anxiety and depression among Chinese pulmonary tuberculosis (PTB) patients, especially those with tracheobronchial tuberculosis (TBTB). The goal of the present study was to explore the prevalence of and associated factors of anxiety and depressive symptoms among PTB patients with and without TBTB.

**Methods:** A cross-sectional survey of PTB patients from three hospitals in Liaoning, China, was conducted using a structured questionnaire. Depression and anxiety were evaluated by using the Hospital Anxiety and Depression Scale (HADS) and the Patient Health Questionnaire-9 (PHQ-9).

**Results:** According to HADS and PHQ-9, 17.73 and 18.13% of 1252 PTB patients, respectively, had significant depressive symptoms and based on HADS scale, 18.37% had significant anxiety symptoms. Approximately 70% of patients with probable depression also had significant anxiety symptoms, and vice versa, and 69.6% patients with anxiety symptoms were also diagnosed with probable depression in our study population. Dyspnea and TBTB were significantly associated with depressive symptoms. Other depressive symptoms-related factors included age, divorce, abnormal body mass index (BMI), and low income. Patients with lower incomes, symptoms of dyspnea, or a combination of ≥3 clinical symptoms had an increased risk of anxiety symptoms, while patients with occasional or frequent alcohol consumption had a reduced risk of anxiety symptoms.

**Conclusion:** Depressive and anxiety symptoms are common among PTB patients, especially those with TBTB. Screening for depression and anxiety is essential for identifying patients who require further psychosocial assessment and support.

## Introduction

Tuberculosis (TB) remains one of the world's deadliest communicable diseases. In 2014, ~9.6 million people (5.4 million men, 3.2 million women, and 1.0 million children) developed TB, and 1.5 million (890,000 men, 480,000 women and 140,000 children) died from TB. China has both a high TB burden and a high multidrug-resistant TB burden^1^. Patients with TB may suffer from mental disorders as a result of long-term treatment, anti-TB drug side effects and TB relapses (1, 2). Anxiety and depression are common mental disorders and global public health concerns. The World Health Organization (WHO) estimates that more than 350 million people suffer from depression and that almost 1.0 million people take their own lives each year^2^. However, most people with mental health problems who live in low- or middle-income countries are undiagnosed or improperly treated (1, 3). It is well known that the prevalence of depression is high among people with chronic diseases (4–8), and several studies have reported that psychiatric morbidities are common in TB patients (1, 2, 9, 10). Depression is associated with poor treatment adherence in patients with chronic diseases. Poor treatment adherence may lead to irregular treatment and lower treatment success rates (6). Previous studies reported that the prevalence of depression in patients with pulmonary tuberculosis (ranging from 13.5 to 72%) was higher than that in the general population (2, 11–15). However, little is known about the prevalence of anxiety and depression among TB patients in China, and little is known about whether tracheobronchial tuberculosis (TBTB) influences symptoms of anxiety or depression. Studies also suggested that the identification rate of depression was underestimated in a general hospital in China (16). The present study aimed to evaluate the prevalence of and factors associated with depressive and anxiety symptoms among TB patients with and without TBTB in Liaoning Province, China and to evaluate whether TBTB is a risk factor for depressive symptoms and/or anxiety symptoms.

## Methods

### Study design

A cross-sectional survey was conducted to describe anxiety and depression states and the social factors among pulmonary tuberculosis (PTB) patients with and without TBTB.

### Study site

This study was conducted in three hospitals in Liaoning Province, China, that care for patients with TB.

### Subjects

We recruited PTB patients who were inpatients and newly diagnosed or were undergoing treatment via directly observed therapy (DOT) between May 2014 and October 2015 for this study. All patients underwent bronchoscopy. Patients with drug resistance; patients with simple extra-pulmonary TB without PTB; patients aged < 18 years; patients who were pregnant, were critically ill, or had communication problems; and patients with severe illnesses, such as cancer, were excluded from this study. We finally enrolled 1252 patients in the study. All cases of PTB were diagnosed according to microbiological testing, and all diagnoses of tracheobronchial tuberculosis were confirmed via bronchoscopy. The diagnosis of TBTB was confirmed via bronchoscopy and histology, as well as microbiological testing (tissue biopsy and/or brushing sample cultures for MTB) (17). We divided patients with TBTB into 7 subtypes based on bronchoscopic manifestations: caseating, edematous-hyperemic, fibrostenotic, granular, tumorous, ulcerative, and non-specific bronchitis (17). Additionally, airway stenosis was classified into five grades according to the decrease in the cross-sectional area of the airway: grade 1(< 25%), grade 2 (25–50%), grade 3 (50–75%), grade 4 (75–90%), and grade 5 (90–100%) (18).

### Data collection

Consecutive PTB patients admitted to three hospitals who met our inclusion criteria were enrolled in this study. Data were collected using a structured questionnaire administered by data collectors. These data collectors were doctors practicing at the abovementioned hospitals and were provided uniform training to conduct standardized interviews and collect data. The structured questionnaire was designed to collect information regarding socio-demographic factors, such as demographics, body mass index (BMI), educational level, occupation status, marital status, monthly income, family history, smoking status, alcohol consumption, comorbidities, TB symptoms, and other social factors. The Hospital Anxiety and Depression Scale (HADS) and the Patient Health Questionnaire-9 (PHQ-9) were included in the questionnaire to evaluate patient depressive and anxiety symptoms. The modified Medical Research Council (mMRC) Dyspnea Scale was used to evaluate dyspnea severity. All the scales were the Chinese versions, which were valid and efficient tools for screening for depression and anxiety (19–21). Patient information pertaining to treatment was collected from the medical databases of the health facilities to which the patients were admitted.

### HADS

HADS is a questionnaire comprising 14 items that is used to screen for symptoms of anxiety and depression. Subscales comprising seven items were used to evaluate both anxiety and depression. Scores of 8 or above on the anxiety or depression subscale were considered indicative of probable “caseness” for either disorder (22).

### PHQ-9

Depression was assessed using the nine-item PHQ-9, a self-administered version of the PRIME-MD diagnostic instrument for common mental disorders. The PHQ-9 consists of 9 questions based on the DSM-IV diagnostic criteria for depressive disorders. Each of these nine items can be scored as 0 (not at all), 1 (several days), 2 (more than half of the days), or 3 (nearly every day). The total score on the questionnaire ranges from 0 to 27. PHQ-9 scores can be dichotomized using a cut-off score of 10, which has a high sensitivity and specificity for the diagnosis of depression (23, 24). The PHQ-9 tool has been demonstrated to be widely useful for assessing patients, including TB patients, for depressive symptoms (25–27). Thus, PHQ-9 scores of 10 or above are considered indicative of probable “caseness” for depression.

### mMRC dyspnea scale

We assessed dyspnea severity using the mMRC dyspnea scale, which consists of five dyspnea severity grades ranging from none (grade 0) to almost complete incapacity (grade 4): Grade 0 (I only get breathless with strenuous exercise); Grade 1 (I get short of breath when hurrying on the level or walking up a slight hill); Grade 2 (I walk slower than people of the same age on the level because of breathlessness, or I have to stop for breath when walking on my own pace on the level); Grade 3 (I stop for breath after walking ~100 meters or after a few minutes on the level); Grade 4 (I am too breathless to leave the house or I am breathless when dressing or undressing) (28).

### Statistical analysis

Normally distributed data are reported as the means ± standard deviation, and comparisons between groups were conducted using Student's *t* test or the Mann-Whitney U test as appropriate. χ^2^-tests were used to compare proportions between groups. Odds ratios (ORs) pertaining to the relationships between risk factors and outcomes (anxiety or depression) along with their corresponding 95% confidence intervals (95% CIs) were calculated. Variables that were considered risk factors for anxiety and depression (*p* < 0.1) were entered into a multivariate logistic regression model to calculate the ORs and their confidence intervals. *P*-values < 0.05 were considered significant. All statistical analyses were performed using Statistical Package for Social Sciences (SPSS) version 17.0 for Windows (SPSS Institute Inc., Chicago, Illinois, USA).

### Ethical considerations

Ethical approval was obtained from the Institutional Ethical Review Board of the First Hospital of China Medical University, Shenyang, China. In addition, research permission was obtained from each hospital. All patients provided written informed consent to participate in this study. We suggested that the patients with depressive and anxiety symptoms undergo further evaluation at the department of mental disease.

## Results

### Socioeconomic and demographic characteristics

Of the PTB patients enrolled in this study, 739 (59.03%) were male, and 513 (40.97%) were female. The mean age at diagnosis was 44.35 years. Approximately 57.91% of patients exhibited more than 4 symptoms, and 217 (17.33%) patients had TBTB. A total of 212 (16.93%) patients had a low BMI < 18.5 kg/m^2^ and 79 (6.31%) patients had a high BMI (>25 kg/m^2^). All the patient characteristics are shown in Table 1.

**Table 1:** Characteristics of PTB patients.

**Characteristic**	**Mean ±*SD***	***n***	**(%)**
Age, years	44.35 ± 17.36		
18–30		380	30.35
31–50		371	29.63
51–80		466	37.22
>80		35	2.80
**SEX**			
Male		739	59.03
Female		513	40.97
BMI, kg/m^2^	21.37 ± 3.48		
< 18.5		212	16.93
18.5 – 25		958	76.52
>25		79	6.31
**MONTHLY INCOME, YUAN**			
< 1,500		319	25.48
1,500-3,000		555	44.33
3,000-5,000		279	22.28
5,000-1,000		47	3.75
>10,000		13	1.04
**MARITAL STATUS**			
Single		347	27.72
Married		845	67.49
Divorced		40	3.19
Widowed		19	1.52
**EDUCATION LEVEL**			
Elementary		121	9.66
Middle school		429	34.27
High school		341	27.24
Vocational/college		332	26.52
Undergraduate		16	1.28
None		12	0.96
**ALCOHOL CONSUMPTION**			
Never drinker		711	56.79
Occasional drinker		367	29.31
Frequent drinker		174	13.90
**SMOKING**			
Never smoker		830	66.29
Current smoker		283	22.60
Former smoker		139	11.10
**MD FAMILY**			
No		1,248	99.68
Yes		4	0.32
**TB FAMILY**			
No		1,191	95.13
Yes		61	4.87
**DUST EXPOSURE**			
No		1,166	93.13
Yes		64	5.11
**OCCUPATION STATUS**			
Unemployed		728	57.41
Employed		534	42.11
**mMRC DYSPNEA SCORE**			
0		699	55.83
1		356	28.43
2		125	9.98
3		49	3.91
4		23	1.84
**SYMPTOMS** ≥**4**			
No		527	42.09
Yes		725	57.91
**COPD**			
No		1,224	97.76
Yes		28	2.24
**TRACHEOBRONCHIAL TUBERCULOSIS**			
No		1,035	82.67
Yes		217	17.33

*PTB, pulmonary tuberculosis; BMI, body mass index; MD, mental disorder; TB, tuberculosis; mMRC, modified Medical Research Council*.

### Prevalence of depressive and anxiety symptoms among TB patients

A total of 222 (17.73%) PTB patients were found to have depressive symptoms according to the PHQ-9, and 227 (18.13%) PTB patients were found to have depressive symptoms according to the HADS. The prevalence of probable depression, as determined by the above scales, was similar (*p* > 0.05). A total of 230 (18.37%) PTB patients were found to have anxiety symptoms according to HADS. A total of 160 (12.78%) PTB patients had both depressive and anxiety symptoms and had a high mMRC dyspnea score compared to those with depressive symptoms or anxiety symptoms only (Table 2).

**Table 2:** Comparative analysis of TB patients with different depression or anxiety status by HADS.

**Demographic characteristic**	**Probable depression only by PHQ-9**	**Probable anxiety only by HADS-A**	**Probable depression only by HADS-D**	**Probable depression and anxiety by HADS-A+HADS-D**
	***n*(%) N = 222**	***n*(%) N = 70**	***n*(%) N = 67**	***n*(%) N = 160**
**AGE, YEARS**				
18–30	54 (24.32)	25 (35.71)	12 (17.91)	41 (25.63)
31–50	71 (31.98)	20 (28.57)	23 (34.33)	49 (30.63)
51–80	89 (40.09)	24 (34.29)	31 (46.27)	65 (40.63)
81–88	8 (3.6)	1 (1.43)	1 (1.49)	5 (3.13)
**SEX**				
Male	136 (61.26)	38 (54.29)	48 (71.64)	95 (59.38)
Female	86 (38.74)	32 (45.71)	19 (28.36)	65 (40.63)
**BMI, KG/M**^2^				
< 18.5	55 (24.77)	14 (20)	11 (16.42)	41 (25.63)
≥18.5, < 25	151 (68.02)	46 (65.71)	52 (77.61)	107 (66.88)
≥25	16 (7.21)	10 (14.29)	4 (5.97)	12 (7.5)
**MONTHLY INCOME, YUAN**				
< 1,500	73 (33.95)	18 (27.27)	24 (36.92)	55 (35.26)
≥1,500	142 (66.05)	48 (72.73)	41 (63.08)	101 (64.74)
mMRC DYSPNEA SCORE*				
0	51 (22.97)	25 (35.71)	29 (43.28)	38 (23.75)
1	59 (26.58)	25 (35.71)	21 (31.34)	37 (23.13)
2	60 (27.03)	19 (27.14)	12 (17.91)	37 (23.13)
3	32 (14.41)	1 (1.43)	4 (5.97)	34 (21.25)
4	20 (9.01)	0 (0)	1 (1.49)	14 (8.75)
**TRACHEOBRONCHIAL TUBERCULOSIS**				
No	169 (76.13)	52 (74.29)	52 (77.61)	125 (78.13)
Yes	53 (23.87)	18 (25.71)	15 (22.39)	35 (21.88)

*Differences in groups according to a significant result from a Kruskal–Wallis test. Patients with both probable depression and probable anxiety had a high mMRC dyspnea score compared to those with probable depression or probable anxiety only.

### Factors associated with depressive symptoms according to the different diagnostic scales

According to a multivariate analysis, a PHQ-9 score >10 was associated with older age, divorce, abnormal BMI, dyspnea, and TBTB. Protective factors (according to a multivariate analysis) included normal BMI (18.5 ≤ BMI < 25), monthly income ≥1,500 Yuan (RMB), higher education level (vocational/college/undergraduate) and employment (Table 3).

**Table 3:** Multivariate analysis for probable depression or anxiety among PTB patients.

**Demographic characteristic**	**PHQ-9(P& aOR[95%CI])**		**HADS-D (P& aOR[95%CI])**		**HADS-A(P& aOR[95%CI])**	
**AGE, YEARS**						
18–30		1.000		1.000		1.000
31–50	0.048	0.55 (0.304, 0.994)	0.734	0.907 (0.515, 1.596)	0.219	0.702 (0.399, 1.234)
51–80	0.023	0.48 (0.256, 0.902)	0.736	0.901 (0.49, 1.657)	0.340	0.746 (0.408, 1.363)
81–88	0.093	0.336 (0.094, 1.199)	0.158	0.401 (0.113, 1.427)	0.110	0.349 (0.096, 1.27)
**SEX**						
Male		1.000		1.000		1.000
Female	0.865	0.964 (0.63, 1.475)	0.522	0.877 (0.587, 1.311)	0.759	1.064 (0.716, 1.58)
**BMI, KG/M**^2^						
< 18.5		1.000		1.000		1.000
≥18.5, < 25	0.010	0.561 (0.362, 0.871)	0.147	0.73 (0.478, 1.116)	0.049	0.66 (0.436, 0.999)
≥25	0.549	0.791 (0.368, 1.702)	0.983	1.008 (0.486, 2.091)	0.312	1.427 (0.717, 2.84)
**MONTHLY INCOME**						
< Y 1,500 yuan		1.000		1.000		1.000
≥ Y 1,500 yuan	0.061	0.683 (0.459, 1.018)	0.007	0.602 (0.416, 0.871)	0.427	0.86 (0.593, 1.247)
**MARITAL STATUS**						
Single		1.000		1.000		1.000
Married	0.069	1.692 (0.96, 2.983)	0.928	0.975 (0.571, 1.668)	0.962	0.987 (0.58, 1.679)
Divorced	0.007	4.018 (1.47, 10.983)	0.461	1.436 (0.549, 3.757)	0.008	3.425 (1.375, 8.532)
Widowed	0.703	1.343 (0.296, 6.088)	0.638	0.698 (0.156, 3.124)	0.872	0.882 (0.192, 4.064)
**EDUCATION LEVEL**						
None/Elementary school		1.000		1.000		1.000
Middle /High school	0.969	1.011 (0.569, 1.798)	0.869	0.956 (0.561, 1.63)	0.891	1.039 (0.598, 1.807)
Vocational/college/ Undergraduate	0.233	0.642 (0.31, 1.33)	0.095	0.557 (0.281, 1.106)	0.776	0.905 (0.456, 1.796)
**ALCOHOL DRINKING**						
Never drinker						
Occasional drinker						
Frequent drinker						
**SMOKING**						
Never smoker		1.000		1.000		1.000
Current smoker	0.108	1.456 (0.921, 2.303)	0.102	1.434 (0.931, 2.209)	0.362	1.229 (0.789, 1.913)
Former smoker	0.257	1.4 (0.783, 2.503)	0.473	1.221 (0.707, 2.11)	0.290	1.348 (0.775, 2.342)
**MD FAMILY**						
No						
Yes						
**TB FAMILY**						
No		1.000		1.000		1.000
Yes	0.595	1.222 (0.584, 2.553)	0.759	1.12 (0.542, 2.316)	0.882	1.055 (0.522, 2.129)
**DM**						
No		1.000		1.000		
Yes	0.072	1.535 (0.962, 2.449)	0.594	1.13 (0.722, 1.768)		
**HBP**						
No						
Yes						
**COPD**						
No		1.000		1.000		1.000
Yes	0.382	0.639 (0.234, 1.743)	0.826	1.11 (0.438, 2.812)	0.501	0.715 (0.268, 1.903)
**DUST EXPOSURE**						
No		1.000		1.000		
Yes	0.108	1.779 (0.882, 3.591)	0.408	1.338 (0.672, 2.663)		
**OCCUPATION STATUS**						
Unemployed		1.000		1.000		1.000
Employed	0.970	0.993 (0.674, 1.463)	0.810	0.956 (0.662, 1.381)	0.008	0.606 (0.418, 0.878)
**mMRC SCORE**						
0						1.000
1	0.000	2.332 (1.525, 3.568)	0.011	1.662 (1.121, 2.463)	0.002	1.904 (1.279, 2.835)
2	0.000	12.512 (7.497, 20.879)	0.000	5.537 (3.407, 8.998)	0.000	7.526 (4.639, 12.211)
3	0.000	28.781 (14.104, 58.732)	0.000	31.527 (14.862, 66.882)	0.000	30.952 (14.974, 63.977)
4	0.000	87.322 (21.822, 349.425)	0.000	14.37 (5.221, 39.548)	0.000	14.718 (5.407, 40.064)
**SYMPTOMS** ≥**4**						
No		1.000		1.000		1.000
Yes	0.567	0.895 (0.612, 1.309)	0.502	0.886 (0.622, 1.262)	0.608	1.099 (0.767, 1.575)
**TRACHEOBRONCHIAL TUBERCULOSIS**						
No		1.000		1.000		1.000
Yes	0.008	1.944 (1.188, 3.18)	0.004	1.991 (1.25, 3.171)	0.025	1.68 (1.067, 2.645)

*PHQ-9, Patient Health Questionnaire-9 (depression: PHQ-9≥10); HADS, Hospital Anxiety and Depression Scale (anxiety /depression: HADS≥8); PTB, pulmonary tuberculosis; BMI, body mass index; MD, mental disorder; TB, tuberculosis; DM, diabetes mellitus; HBP, high blood pressure; COPD, chronic obstructive pulmonary disease; mMRC, modified Medical Research Council*.

When depressive symptoms were assessed using the HADS-D, only dyspnea and TBTB remained as risk factors, as well as a monthly income < 1,500 Yuan (RMB). Common protective factors (according to a multivariate analysis) included monthly income ≥1,500 Yuan (RMB) (Table 3).

The potential risk factors for anxiety symptoms were examined using multivariate models. Patients suffering from high-grade dyspnea, as determined using the mMRC dyspnea scale, were at increased risk for anxiety symptoms. Moreover, normal BMI and employment were protective factors against anxiety symptoms among PTB patients. The results of this analysis are presented in Table 3.

### Comparative analysis of TB patients with and without TBTB

Two depression screening scales (HADS and PHQ-9) demonstrated that TB patients with TBTB were more likely to suffer from probable depression than were TB patients without TBTB. The ORs pertaining to the risk of depressive symptoms in these groups of patients were 1.451 and 1.656, respectively. Moreover, TBTB was significantly related to anxiety symptoms, according to the HADS (Table 4).

**Table 4:** Comparative analysis of TB patients with or without TBTB.

	**With TBTB (*****n*** = **217)**		**Without TBTB (*****n*** = **1,035)**					
	**N**	**%**	**N**	**%**	***p***	**OR**	**95%CI**	
Anxiety symptoms (HADS)	53	24.42	177	17.10	0.011	1.567	1.105	2.222
Depression Symptoms(HADS)	50	23.04	177	17.10	0.039	1.451	1.018	2.070
Depressive symptoms(PHQ-9)	21	9.68	50	4.83	0.005	2.111	1.240	3.594
**mMRC DYSPNEA SCORE**								
0	114	52.53	585	56.52	–			
1	65	29.95	291	28.12	0.425	1.146	0.820	1.603
2	26	11.98	99	9.57	0.219	1.348	0.837	2.170
3	7	3.23	42	4.06	0.710	0.855	0.375	1.951
4	5	2.30	18	1.74	0.492	1.425	0.519	3.917

*TB, tuberculosis; TBTB, tracheobronchial tuberculosis; HADS, Hospital Anxiety and Depression Scale; PHQ-9, Patient Health Questionnaire; mMRC, modified Medical Research Council*.

### The impact of different characteristics of TBTB on the prevalence of depressive and anxiety symptoms

We grouped TBTB patients according to the different characteristics and compared the incidence of depressive and anxiety symptoms evaluated by the PHQ9 and HADS (Table 5). No difference was found when comparing the incidence of depressive and anxiety symptoms in patients with different of bronchoscopic subtypes (*p* > 0.05). Additionally, a comparison of TBTB patients with tracheal/bronchial involvement with those without central airway involvement showed that the incidence of depressive and anxiety symptoms was not significantly different between the two groups (*p* > 0.05). Our results revealed that, among TBTB patients, those with grade 4 stenosis had a higher incidence of depressive and anxiety symptoms than those with grade 1–3 stenosis (*p* = 0.005; 0.005; 0.023). The patient with grade 5 stenosis was not included because our sample did not contained case with grade 5 stenosis.

**Table 5:** Comparative analysis of TB patients with different characteristics of TBTB.

		**PHQ9**		**HADS-A**		**HADS_D**	
		***n*** **(%)**	χ**2*****/p***	***n*****(%)**	χ**2*****/p***	***n*** **(%)**	χ**2*****/p***
Bronchoscopic subtype	Fibrostenotic	15/58 (25.86)	5.891/0.317	13/58 (22.41)	5.061/0.408	15/58 (25.86)	6.075/0.299
	Edematous-hyperemic	16/70 (22.86)		17/70 (24.29)		17/70 (24.29)	
	Granular	0/11 (0)		1/11 (9.09)		1/11 (9.09)	
	Tumorous	2/5 (40)		3/5 (60)		3/5 (60)	
	Caseating	18/69 (26.09)		18/69 (26.09)		13/69 (18.84)	
	Ulcerative	2/4 (50)		1/4 (25)		1/4 (25)	
	Total	53/217 (24.42)		53/217 (24.42)		50/217 (23.04)	
Involved site	Non-trachea/main bronchus	32/141 (22.7)	0.652/0.419	33/141 (23.4)	0.227/0.634	33/141 (23.4)	0.030/0.863
	Trachea/main bronchus	21/76 (27.63)		20/76 (26.32)		17/76 (22.37)	
	Total	53/217 (24.42)		53/217 (24.42)		50/217 (23.04)	
Stenosis grading	1	14/57 (24.56)	8.691/0.034, 7.913/0.005*	17/57 (29.82)	16.09/0.001, 7.913/0.005*	19/57 (33.33)	14.675/0.002, 5.206/0.023*
	2	14/74 (18.92)		9/74 (12.16)		7/74 (9.46)	
	3	18/74 (24.32)		20/74 (27.03)		18/74 (24.32)	
	4	7/12 (58.33)		7/12 (58.33)		6/12 (50)	
	Total	53/217 (24.42)		53/217 (24.42)		50/217 (23.04)	

*Comparison of Grade 4 cases with the combined set of Grade 1-3 cases.

### The impact of interventional therapy on the prevalence of depressive and anxiety symptoms among TBTB patients with central airway involvement

Among TBTB patients with central airway involvement, treated patients had a lower incidence of depressive and anxiety symptoms than those in the untreated group (*p* = 0.001; 0.007; < 0.001; Table 6). Interventional therapy may be a protective factor against depressive and anxiety symptoms.

**Table 6:** The impact of Interventional therapy on prevalence of depression and anxiety symptoms among TBTB patients with central airway involvement.

	**Untreated group *n* (%)**	**Treated group *n* (%)**	***p***	**OR (95%CI)**
Anxiety Symptoms(HADS)	15/32(46.88)	5/44(11.36)	0.001	0.145(0.045, 0.464)
Depression Symptoms(HADS)	12/32(37.5)	5/44(11.36)	0.007	0.214(0.066, 0.691)
Depression Symptoms(PHQ-9)	16/32(50)	5/44(11.36)	0.000	0.128(0.04, 0.409)

## Discussion

In recent years, the psychiatric status of patients with TB has attracted increasing attention because psychiatric illnesses such as anxiety and depression have been confirmed to be associated with poor treatment outcomes in TB patients (1, 29). Unfortunately, there remains a lack of medical literature regarding the prevalence of depressive and anxiety symptoms among PTB patients in China, especially patients with TBTB. Our study included many PTB patients living in Liaoning Province, China. We and our colleagues prospectively administered structured questionnaires to 1252 patients with PTB to evaluate the prevalence of depressive and anxiety symptoms among PTB patients.

The prevalence of probable depression in this PTB population was 17.73% according to the PHQ-9 and 18.13% according to the HADS. Other studies determined that the prevalence of depression among patients with TB ranged from 16.8% in the Philippines to 80% among hospitalized patients in Pakistan (30, 31). Compared with the prevalence of depression noted among patients enrolled in previous studies, the prevalence of probable depression noted among the PTB patients enrolled in the present study was relatively low. One possible explanation for this result is that only the prevalence of major depression was assessed in the present study, whereas other mental disorders were assessed in some previous studies. The abovementioned difference may also have resulted from differences in the races, countries and patient populations evaluated in this study compared with those evaluated in previous studies (the prevalence of depression was highest in hospitalized patients). Our study also noted a non-significant difference in the prevalence of depressive symptoms determined using the PHQ-9 and HADS and determined that the risk factors for depressive symptoms were different when probable depression was diagnosed using different scales.

In this study, we determined that young age (18–30 years) and divorced marital status may increase the risk of probable depression in TB patients. The increased risk of depressive symptoms noted among young people may be attributable to social pressures and feelings of shame spurred by diagnosis with TB. Patients who are divorced tend to be lonely and have less social support than patients who are married, thus, marital status may be related to depressive symptoms (9, 32). However, age had no independent effect when we used the HADS scale. We also determined that dyspnea and large numbers of clinical symptoms (≥4) were associated with depressive symptoms among PTB patients. Previous studies demonstrated that multiple symptoms and dyspnea are risk factors for depression not only among PTB patients (31) but also among patients with chronic respiratory diseases, such as COPD and asthma (11, 33). Regarding monthly income, PTB patients with high income levels were less likely to suffer from depressive and anxiety symptoms than were patients with low income levels (9, 15, 34). This study also demonstrated that smoking history was correlated with depressive symptoms among PTB patients. Smoking is also a risk factor for depression in the general population (1, 35).

Interestingly, alcohol consumption, whether occasional or frequent, was associated with a reduced risk of anxiety symptoms. In our study, patients usually did not meet the criteria for the diagnosis of alcohol abuse. Perhaps the negative coping of these patients through alcohol consumption served to ameliorate anxiety in the short term. A study focusing on the longitudinal associations between different levels of alcohol consumption and new-onset depression and generalized anxiety disorder demonstrated that light-to-moderate alcohol consumption was a protective factor against depression and generalized anxiety. The authors of that study attributed their findings to the antidepressant and anxiolytic effects of moderate alcohol use. However, the exact mechanism underlying the effects of alcohol on depression is largely unknown (36).

Finally, our PHQ-9 and HADS results indicated that PTB patients with TBTB were more likely to suffer from probable depression than PTB patients without TBTB. This is the first report to focus on the effects of TBTB on the prevalence of probable depression among PTB patients. A previous study noted that 15 to 90% of patients with TBTB have some degree of bronchial stenosis (37, 38). Thus, the high prevalence of probable depression noted among TBTB patients may be associated with the dyspnea caused by bronchial stenosis. Our finding that patients with the higher severity of stenosis (grade 4 stenosis) had the highest incidence of depressive and anxiety symptoms among TBTB patients supports this hypothesis. On the other hand, the high prevalence of probable depression noted among TBTB patients may be associated with some consistent symptoms such as non-productive cough, which is more common in patients with TBTB than patients with PTB. However, in our study, neither the site of airway involvement nor the bronchoscopic subtypes of TBTB were associated with significant differences in the incidence of depressive and anxiety symptoms. Further research including a larger sample size should be performed to better understand the association between TBTB and depression.

In addition, interventional pulmonology treatments for TBTB may be associated with mental health. We found that treated patients had a lower incidence of anxiety and depressive symptoms than the untreated group among TBTB patients with central airway involvement, which may result from the fact that the interventional treatment can relieve the symptoms of dyspnea.

Additional studies regarding the risk factors for depressive symptoms among TBTB patients are necessary. Once the diagnosis of TBTB has been confirmed, clinicians should pay attention to the mental statuses of patients and assess patients for symptoms of anxiety and depression.

Some limitations of this study should be mentioned. First, as this was a cross-sectional study, we cannot exclude the possibility that our results were affected by recall bias such as a family history of mental disorders and tuberculosis which may under-estimate those factors. Furthermore, a comparison of our results to healthy controls was not possible due to the absence of a control group. Because not all the participated hospitals having department of mental disorder and only used screening questionnaires for anxiety and depression, our study only evaluated the prevalence and risk factors of probable anxiety and depression. But our study still offered valuable information for further study in this field. Finally, cross-sectional surveys can demonstrate associations but cannot demonstrate temporal sequences of events, which may raise concerns regarding the directions of causality between risk factors and depression in patients with TB (39).

## Conclusion

Our study determined the prevalence of and factors associated with depressive and anxiety symptoms among PTB patients in Liaoning Province, China. As depressive and anxiety symptoms are common among PTB patients, especially those with TBTB, screening for depression and anxiety can identify patients who require further psychosocial assessment, support and treatment to experience a better clinical response to anti-TB treatment.

## Author contributions

GH: Substantial contributions to the conception or design of the work, Interpretation of data for the work; XW and XL: Analysis of data for the work; GH, XW, and XL: Drafting the work; All the authors: Acquisition of data for the work, Revising the work critically for important intellectual content, Final approval of the version to be published, Agreement to be accountable for all aspects of the work in ensuring that questions related to the accuracy or integrity of any part of the work are appropriately investigated and resolved.

### Conflict of interest statement

The authors declare that the research was conducted in the absence of any commercial or financial relationships that could be construed as a potential conflict of interest.

## Footnotes

^1^http://apps.who.int/iris/bitstream/10665/191102/1/9789241565059eng.pdf.

^2^http://www.who.int/mediacentre/factsheets/fs369/en/.

## Abbreviations

PTB: pulmonary tuberculosis
TBTB: tracheobronchial tuberculosis
HADS: Hospital Anxiety and Depression Scale
PHQ-9: Patient Health Questionnaire-9
TB: tuberculosis
DOT: directly observed therapy
mMRC dyspnea scale: modified Medical Research Council dyspnea scale
ORs: odds ratios
95% CIs: 95% confidence intervals
BMI: body mass index
DM: diabetes mellitus
COPD: chronic obstructive pulmonary disease.
